# HDV Can Constrain HBV Genetic Evolution in HBsAg: Implications for the Identification of Innovative Pharmacological Targets

**DOI:** 10.3390/v10070363

**Published:** 2018-07-09

**Authors:** Luna Colagrossi, Romina Salpini, Rossana Scutari, Luca Carioti, Arianna Battisti, Lorenzo Piermatteo, Ada Bertoli, Lavinia Fabeni, Carmine Minichini, Pascale Trimoulet, Hervé Fleury, Elena Nebuloso, Maria De Cristofaro, Giuseppina Cappiello, Alberto Spanò, Vincenzo Malagnino, Terenzio Mari, Angelo Barlattani, Nerio Iapadre, Miriam Lichtner, Claudio Mastroianni, Ilaria Lenci, Caterina Pasquazzi, Giuseppe Maria De Sanctis, Alfonso Galeota Lanza, Maria Stanzione, Gianfranca Stornaiuolo, Massimo Marignani, Loredana Sarmati, Massimo Andreoni, Mario Angelico, Francesca Ceccherini-Silberstein, Carlo-Federico Perno, Nicola Coppola, Valentina Svicher

**Affiliations:** 1 Department of Experimental Medicine and Surgery, Tor Vergata University, 00133 Rome, Italy; luna_colagrossi@yahoo.it (L.C.); rsalpini@yahoo.it (R.S.); scutari.rossana@gmail.com (R.S.); luca.carioti@yahoo.com (L.C.); battisti.arianna@gmail.com (A.B.); piermatteolorenzo@gmail.com (L.P.); bertoli@uniroma2.it (A.B.); lavinia.fabeni@gmail.com (L.F.); ceccherini@med.uniroma2.it (F.C.-S.); carlo.perno@unimi.it (C.-F.P.); 2 Department of Mental Health and Public Medicine, Section of Infectious Diseases, University of Campania L. Vanvitelli, 81100 Naples, Italy; carmine.minichini@alice.it (C.M.); nicola.coppola@unina2.it (N.C.); 3 Laboratoire de Virologie, Hôpital Pellegrin tripode, 33076 Bordeaux, France; pascale.trimoulet@chu-bordeaux.fr (P.T.); herve.fleury@u-bordeaux.fr (H.F.); 4 Unit of Microbiology, Sandro Pertini Hospital, 00157 Rome, Italy; elena.nebuloso@aslromab.it (E.N.); maria.decristofaro@aslroma2.it (M.D.C.); giuseppina.cappiello@aslroma2.it (G.C.); alberto.spano@aslromab.it (A.S.); 5 Infectious Diseases Unit, Tor Vergata University Hospital, 00133 Rome, Italy; malagninovincenzo@gmail.com (V.M.); sarmati@med.uniroma2.it (L.S.); andreoni@uniroma2.it (M.A.); 6 Hepatology Unit, Nuovo Regina Margherita Hospital, 00153 Rome, Italy; terenziomari@virgilio.it (T.M.); epatologia@barlattani.it (A.B.); 7 Infectious Diseases Unit, San Salvatore Hospital, 67100 L’Aquila, Italy; nerioiapadre@libero.it; 8 Department of Public Health and Infectious Diseases, Sapienza University, 00185 Rome, Italy; miriam.lichtner@gmail.com (M.L.); claudio.mastroianni@uniroma1.it (C.M.); 9 Hepatology Unit, Tor Vergata University Hospital, 00133 Rome, Italy; ilaria.lenci@uniroma2.it (I.L.); angelico@med.uniroma2.it (M.A.); 10 Department of Gastroenterology, S. Andrea Hospital, 33771 Rome, Italy; pasquazzi@yahoo.it; 11 Department of Infectious Diseases, Umberto I Hospital, 00161 Rome, Italy; giuseppemaria.desanctis@uniroma1.it; 12 Gastroenterology Unit, AO Cardarelli, 80131 Naples, Italy; a.galeotalanza@alice.it; 13 Department of Internal Medicine, University of Campania L. Vanvitelli, Viral Unit, 81100 Naples, Italy; marinella.stanzione@inwind.it (M.S.); gstornaiuolo@libero.it (G.S.); 14 Liver Unit, AOU, S. Andrea Hospital, 00189 Rome, Italy; mmarignani@hotmail.com; 15 Haematology and Oncohematology, University of Milan, 20122 Milan, Italy

**Keywords:** HBsAg, HDV-RNA, HDAg

## Abstract

Chronic HBV + HDV infection is associated with greater risk of liver fibrosis, earlier hepatic decompensation, and liver cirrhosis hepatocellular carcinoma compared to HBV mono-infection. However, to-date no direct anti-HDV drugs are available in clinical practice. Here, we identified conserved and variable regions in HBsAg and HDAg domains in HBV + HDV infection, a critical finding for the design of innovative therapeutic agents. The extent of amino-acid variability was measured by Shannon-Entropy (Sn) in HBsAg genotype-d sequences from 31 HBV + HDV infected and 62 HBV mono-infected patients (comparable for demographics and virological-parameters), and in 47 HDAg genotype-1 sequences. Positions with Sn = 0 were defined as conserved. The percentage of conserved HBsAg-positions was significantly higher in HBV + HDV infection than HBV mono-infection (*p* = 0.001). Results were confirmed after stratification for HBeAg-status and patients’ age. A Sn = 0 at specific positions in the C-terminus HBsAg were correlated with higher HDV-RNA, suggesting that conservation of these positions can preserve HDV-fitness. Conversely, HDAg was characterized by a lower percentage of conserved-residues than HBsAg (*p* < 0.001), indicating higher functional plasticity. Furthermore, specific HDAg-mutations were significantly correlated with higher HDV-RNA, suggesting a role in conferring HDV replicative-advantage. Among HDAg-domains, only the virus-assembly signal exhibited a high genetic conservation (75% of conserved-residues). In conclusion, HDV can constrain HBsAg genetic evolution to preserve its fitness. The identification of conserved regions in HDAg poses the basis for designing innovative targets against HDV-infection.

## 1. Introduction

Worldwide, an estimated 15 million individuals with chronic hepatitis B virus (HBV) infection are infected with hepatitis D virus (HDV) [1]. Chronic HBV + HDV infection is associated with a greater risk of liver fibrosis, earlier hepatic decompensation, and liver cirrhosis hepatocellular carcinoma compared to HBV mono-infection [2,3]. Despite the severity of hepatitis D, no direct acting antiviral drugs are so far available in clinical practice, and the diagnostic assays for HDV infection are not fully standardized. Indeed, interferon alpha is the only anti-HDV drug approved for the treatment of chronic hepatitis D, even if its efficacy is limited and side effects can be severe. Future therapeutic options are under investigation targeting HDV entry, HBV surface antigen (HBsAg) secretion, and viral assembly by inhibiting the farnesylation of HDV antigen (HDAg) [4].

So far, eight HDV genotypes have been identified. Except for genotype 1, which is represented worldwide, all other genotypes are mostly found in specific geographical areas [5,6].

HDV is a peculiar virus since it requires HBsAg to infect the hepatocytes [7]. HDV genome is composed by a 1.7 kb single-stranded circular RNA of negative polarity, whose replication requires the involvement of the host RNA polymerase I and II [8,9]. The HDV genome contains only one open reading frame encoding two isoforms of the delta antigen: the small and the large delta antigen (S-HDAg composed by 195 amino acids and L-HDAg composed by 214 amino acids, respectively).

During the replication cycle, the expression of L-HDAg is mediated by host adenosine deaminase (ADAR1) which modifies the stop codon at position 196 to a tryptophan codon in the antigenomic RNA. S-HDAg is involved in the replication of viral genome, while L-HDAg is essential for the assembly of new viral particles by interacting with HBsAg [10].

The S-HDAg contains different functional domains, such as three RNA-binding domains (RBD, aa: 2–27; 97–107; 136–146), a coiled-coil sequence (CCS, aa: 31–67), and a nuclear localization sequence (NLS, aa: 68–88) [11,12]. Beyond the aforementioned domains, L-HDAg contains the virus-assembly signal (VAS, aa: 195–214), which plays a critical role in viral assembly [13].

The virus-assembly signal contains a carboxy-terminal CXXQ (C stands for cysteine, X for any amino acid, and Q for glutamine) sequence for farnesylation [14,15,16]. The farnesyl group has been proposed to anchor the L-HDAg to the endoplasmic reticulum membrane, where HBsAg is synthesized [17]. The interaction between HBsAg and L-HDAg is crucial for HDV morphogenesis, and thus for proper viral particle production. Although previous in vitro studies have investigated mechanisms underlying the process of interaction between the HBsAg and L-HDAg [18,19], a paucity of information is available regarding the extent of genetic variability in HBsAg and HDAg in vivo in patients with HBV + HDV infection. The identification of conserved regions in HDAg and HBsAg is fundamental to identify essential domains crucial for HDV fitness, thus paving the way to identify innovative pharmacological targets to prevent efficient HDV spread in the liver.

In this light, the goals of this study are: (i) to define and compare the degree of genetic variability in HBsAg in the setting of HBV + HDV infection and HBV mono-infection; (ii) to define and characterize variable and conserved regions in HDAg in patients with chronic HBV + HDV infection; (iii) to correlate the extent of HBsAg and HDAg genetic variability with the levels of serum HDV-RNA (reflecting HDV replicative potential in term of HDV particles production).

## 2. Materials and Methods 

### 2.1. Study Population

This study included 78 consecutive patients with chronic HBV + HDV infection, followed in different clinical centers (“Tor Vergata” University Hospital of Rome, Second University of Naples, Hôpital Pellegrin Tripode of Bordeaux, Sandro Pertini Hospital of Rome). In order to provide more readable data on the impact of HBsAg and HDAg genetic variability on serum HDV-RNA, all the 78 patients were naïve to interferon, the only currently available drug capable to affect HDV replication and thus the levels of serum HDV-RNA.

Demographic, clinical, and virological data were collected for each patient and stored in an ad hoc designed anonymous database. Serum HBV-DNA was quantified by Roche COBAS^®^ AmpliPrep/COBAS^®^ TaqMan^®^ HBV Test, v2.0 (Roche Molecular Diagnostics, Pleasanton, CA, USA), while serum HDV-RNA was quantified a commercial HVD Real Time PCR assay (LifeRiver Diagnostics, Shanghai ZJ Bio-Tech Co., Shanghai, China) Shanhai ZK Bio-tech.

Starting from 78 HBV + HDV infected patients, HBsAg genotype D and HDAg genotype 1 sequences were obtained for 31 and 47 patients, respectively. In order to compare HBsAg genetic variability between HBV + HDV infection and HBV mono-infection, HBsAg sequences from 62 patients with HBV genotype D monoinfection were also analyzed. Thirty-one HBV + HDV infected and 62 HBV mono-infected patients were comparable for sex, nationality, age, HBeAg status, HBV-DNA, and anti-HBV drug received.

Ethic approval was deemed unnecessary because, under Italian law, biomedical research is subjected to previous approval by ethics committees only in the hypothesis of clinical trials on medicinal products for clinical use (art. 6 and art. 9, leg. decree 211/2003). The research was conducted on viral DNA or RNA samples (used for clinical routine), and data previously anonymized, according to the requirements set by Italian Data Protection Code (leg. decree 196/2003). Inform consent was not necessary since the study was based on a retrospective analysis of anonymized viral sequences obtained for clinical routine practice.

### 2.2. HBsAg and HDAg Sequencing 

For each patient, HBV-DNA was extracted from plasma sample using a commercially available kit (QIAmp DNA blood mini-kit, Qiagen Inc., Germantown, MD, USA), and then HBsAg (aa:55–226) was amplified with Amplitaq-Gold polymerase using the following primer pairs: HBV_F1-5′GGTCACCATATTCTTGGGAA and HBV_R1-5′GTGGGGGTTGCGTCAGCAAA. Polymerase chain reaction (PCR) conditions were: one cycle at 93 °C for 12 min, 40 cycles (94 °C 50 s, 57 °C 7 50 s, 72 °C 1 min and 30 s), and a final cycle at 72 °C for 10 min. For samples with low serum HBV-DNA, a nested-PCR was performed, starting from the same first amplicon, using the following primer pairs HBV_2D-5′GGTGGACTTCTCTCAATTTT and HBV_7R-TGGCGAGAAAGTGAA. Nested-PCR conditions were: one cycle at 93 °C for 12 min, 30 cycles (94 °C 50 s, 55 °C 50 s, 72 °C 1 min and 20 s), and a final cycle at 72 °C for 10 min.

For HDAg amplification and sequencing, HDV-RNA was extracted using a commercially available kit (QIAmp RNA blood mini-kit, Qiagen Inc., USA), and then amplified with SuperScript III One-Step RT-PCR with Taq Platinum High Fidelity (Invitrogen, Carlsbad, CA, USA) using the following primer pairs: HDV_F1-5′CTTAGCCATCCGAGTGGACG and HDV_R1-5′GTCCAGCAGTCTCCTCTTTACA. PCR conditions were: one cycle at 50 °C for 30 min, one cycle 94 °C for 2 min, 40 cycles (94 °C 15 s, 61 °C 30 s, 68 °C 1 min and 30 s), and a final cycle at 68 °C for 10 min. For samples with low serum HDV-RNA, a nested-PCR was performed starting from the same first amplicon. The PCR-products were amplified with Amplitaq-Gold polymerase using the following primer pairs HDV_F2-5′AGACGCAAACCTGYGAGTGG and HDV_R1 (mentioned before). Nested-PCR conditions were: one cycle at 95 °C for 10 min, 30 cycles (95 °C 20 s, 61 °C 30 s, 72 °C 1 min), and a final cycle at 72 °C for 10 min.

PCR-products were purified and sequenced by using different overlapping sequence-specific primers and a BigDye terminator v. 3.1 cycle sequencing kit (Applied-Biosystems, Foster City, CA, USA).

The sequences were analyzed using SeqScape-v.2.5 software (Thermo Fisher Scientific, Waltham, MA, USA). The quality endpoint for each individual gene was ensured by a coverage of the HBsAg and HDAg sequence by at least two segments. Sequences having a mixture of wild-type and mutant residues at single positions were considered to have the mutant(s) at that position. The mixed base identification was set at a percentage of 20%.

### 2.3. Phylogenetic Analysis

HBV and HDV genotypes were assessed by phylogenetic analysis using HBsAg and HDAg sequences obtained by population-based sequencing. Nucleotide sequences of HBsAg and HDAg were compared to reference sequences representing all known HBV and HDV genotypes retrieved from the Genbank (HBV accession numbers: AB51639, FJ709464, AY090459, DQ899146, DQ899142, AB036920, AF223965, GU563556, FN594748, JN664942, EU594434, GU456636, GQ477452, GQ205377, GQ922003, FN594770, KF170740, FJ904442, AB486012, JN792893, GQ924617, GQ358147, GQ358137, GQ924640, GQ358149, AB642091, FJ899779, GQ924626, FN545833, HE576989, JN182318, GQ331047, AB194951, AY934764, FJ692613, FJ023659, FJ023664, AB644280, AB554025, AB644286, AB554019, AB644287, AB540583, AP011106, HM011493, AB644284, EU410080, EU670263, GU721029, AP011108, GQ358158, AB697490, DQ089801; HDV accession number: X04451, X60193, L22063, AF018077, AJ584848, AJ584847, AJ584844, AJ584849) using MEGA 5.02 (https://www.megasoftware.net/). The phylogenetic analysis was also performed to exclude possible contaminations and to define HBV and HDV genotypes. The phylogenetic trees were generated with the Tamura–Nei model. The statistical robustness within each phylogenetic tree was confirmed with a bootstrap-analysis using 1000 replicates.

### 2.4. Evolutionary Divergence

Shannon Entropy (Sn = −Σi(pi lnpi)/lnN) was calculated to measure the extent of amino acid variability at each HBsAg and HDAg position, where pi was the relative frequency of each distinct amino acid detected at a given position and *n* was the total number of sequences analyzed [20]. For HBsAg, Shannon-Entropy was calculated in the group of 31 HBV + HDV infected and 62 HBV mono-infected patients, and stratifying HBV + HDV infected patients according to serum HDV-RNA (>or <3.5 log serum HDV-RNA). In order to make result more readable, all the patients included in the analysis were infected with HBV genotype D.

For HDAg, Shannon Entropy was calculated on 47 HBV + HDV coinfected patients, and stratifying patients according to serum HDV-RNA (< or >4.5 log serum HDV-RNA).

The thresholds of 3.5 and 4.5 log IU/mL were chosen to stratify the 31 patients with HBsAg sequences available and the 47 patients with HDAg sequences available in two numerically balanced groups. Positions with SE = 0 were defined conserved.

### 2.5. Statistical Analysis

Statistical data were analyzed using SPSS software (v19.0; SPSS Inc., Chicago, IL, USA). Data are expressed as medians (interquartile range [IQR]) for continuous variables, and percentages for discrete variables. The Chi-Squared Test based on a 2 × 2 contingency table was used for dichotomous data while Mann–Whitney for continuous data. *p* < 0.05 was considered as statistically significant.

HBsAg three-dimensional structure prediction. The three-dimensional structure of HBsAg was generated by using I_TASSER following a homology modeling approach with the original HBsAg reference sequence as a modeling template [21,22]. UCSF Chimera software was used to color each HBsAg residue according to its extent of genetic variability [23].

## 3. Results

### 3.1. Patients’ Characteristics

The main epidemiological and virological characteristics of the 78 patients with chronic HBV and HDV infection are reported in Table 1.

Patients were prevalently males (65.4%) with a median (IQR) age of 51 (41–59) years. Serum HBV-DNA was detected in 66 cases (84.6%) with a median (IQR) of 2.0 (1.3–3.1) log IU/mL. Median (IQR) of ALT and AST were 68 (43–127) U/L and 66 (34–101) U/L, respectively. All patients were HDV-RNA positive at qualitative testing. Seventeen patients (21.8%) had a serum HDV-RNA below the lower limit of quantification (<2000 copies/mL). The remaining 61 patients (78.2%) had a median (IQR) serum HDV-RNA of 4.7 (2.5–6.1) log IU/mL).

A negative correlation was observed between serum HBV-DNA and serum HDV-RNA (Rho Spearman = −0.308; *p* value = 0.006).

Among 78 patients, 7 (9.3%) had a diagnosis of HCC, while 20 (28.2%) of cirrhosis (Table 1). Among patients with therapy status available (*n* = 66), 50% (33/66) were exposed to at least one NUC.

Among the HBV + HDV infected patients with detectable serum HBV-DNA, HBsAg sequences were obtained for 42 patients. Phylogenetic analysis revealed that most patients were infected with HBV genotype D (31, 73.8%), followed by HBV genotype A (5, 11.9%), E (5, 11.9%), and B (1, 2.4%). The analysis was focused on 31 patients infected with HBV genotype D, the majority of them were infected with HBV subgenotype D3 (22, 71%), followed by subgenotype D1 (5, 16.1%), D4 (3, 9.7%) and D2 (1, 3.2%). Among patients with serum HDV-RNA >2000 copies/mL, HDAg sequences were obtained for 47 patients, all belonging to HDV genotype 1. No other HDV genotypes were detected.

### 3.2. Characterization of HBsAg Genotype D Genetic Variability in HDV + HBV Infected and HBV Mono-Infected Patients

The degree of HBsAg genetic variability in HBV + HDV infected patients was evaluated on 31 patients infected with HBV genotype D (the most common HBV genotype detected) and was compared to that observed in a group of 62 patients with HBV genotype D mono-infection. HBV mono-infected and HBV + HDV infected patients were comparable for sex, nationality, age, HBeAg status, HBV-DNA, and anti-HBV drug received (Table 2).

Firstly, Shannon Entropy was determined for each HBsAg amino acid position in both HBV + HDV infected and HBV mono-infected patients.

**Figure 1:**
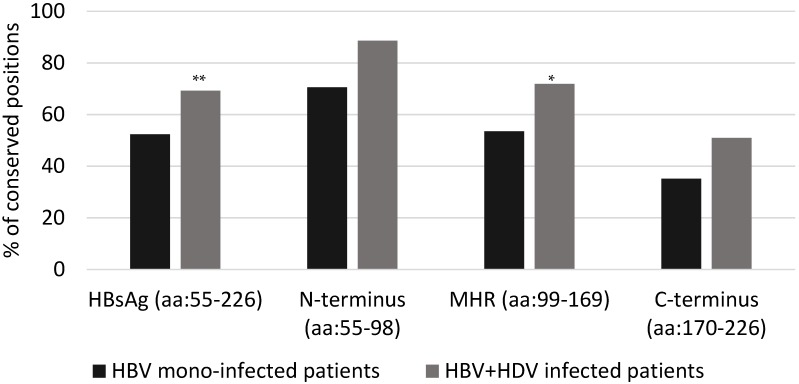
The histogram reports the percentage of conserved HBsAg positions in 31 HBV + HDV coinfected patients (grey bars) and 62 HBV mono-infected patients (black bars). Conserved positions are those with Shannon Entropy = 0. Statistically significant differences were assessed by Chi Square Test. ** *p* = 0.001; * *p* = 0.037.

**Figure 2:**
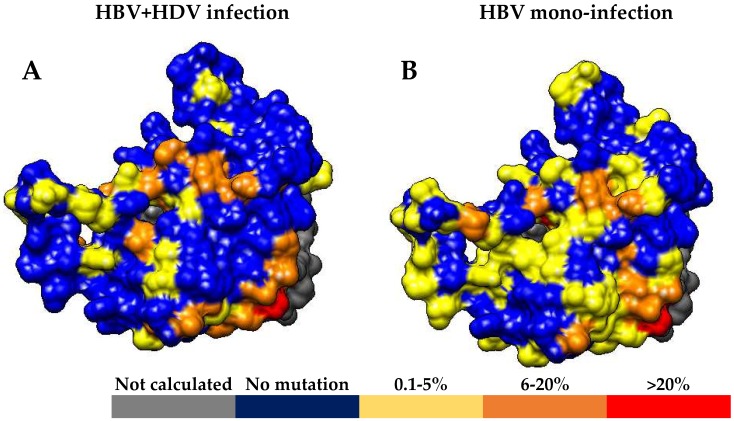
Three-dimensional structure of HBV genotype D HBsAg. HBsAg structure was built by using I-TASSER [24], and colored by using UCSF Chimera [23]. In patients with chronic HBV + HDV infection (**A**) and HBV mono-infection (**B**), residues were colored according to the percentage of mutations detected: 0% Blue, 0.1–5% Yellow, 6–20% Orange and >20% Red.

Mean Shannon Entropy of HBsAg genotype D sequences from HBV + HDV infected patients was significantly lower than that observed for HBsAg sequences from HBV mono-infected patients (mean + SD: 0.07+0.13 for HBV + HDV infection vs. 0.10 + 0.17 for HBV mono-infection, *p* < 0.01). In line with this result, the number of conserved HBsAg positions (Sn = 0) was significantly higher in HBV + HDV infected patients than in HBV mono-infected patients (119/172 [69.2%] vs. 90/172 [52.3%], *p* = 0.001). Comparing the two groups of patients, the percentage of conserved positions was different in each HBsAg domain: 88.6% ([39/44] vs. 70.5% [31/44] (*p* = 0.062) for the N-terminus, 71.8% [51/71] vs. 53.5% [38/71] (*p* = 0.037) for the major hydrophilic region (MHR), and 50.9% [29/57] vs. 35.1% [20/57] (*p* = 0.130) for HBsAg C-terminus (Figure 1 and Figure 2).

**Figure 3:**
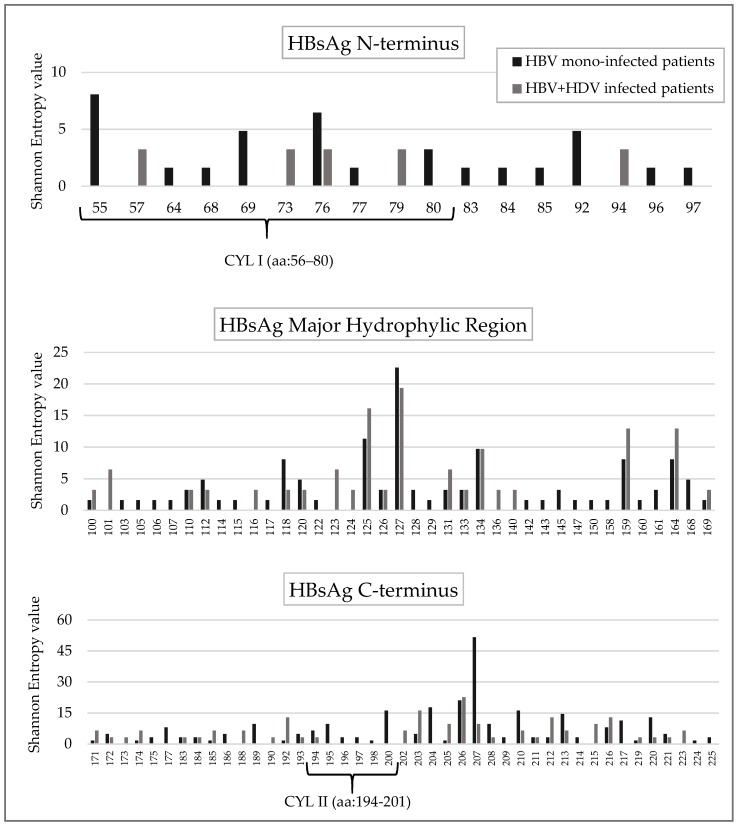
The histogram reports the Shannon Entropy (Sn) value at HBsAg positions in HBV mono-infected patients (black bars) and HBV + HDV infected patients (grey bars). Conserved positions (Sn = 0) in both HBV + HDV infected and HBV mono-infected patients were not reported in the histogram. Shannon Entropy was calculated according to the following formula Sn = −Σi(pi lnpi)/lnN) where pi was the relative frequency of each distinct amino acid detected at a given position and *n* was the total number of sequences analyzed.

Furthermore, specific positions in N-terminus (*n* = 11), MHR (*n* = 19) and C-terminus (*n* = 12) were conserved only in HBV + HDV infected patients (Figure 3 and Figure S1).

The percentage of conserved residues throughout the entire HBsAg genotype D sequences remained higher in HBV + HDV infected patients than in HBV mono-infected patients when the analysis was focused on HBeAg-negative patients (77.9% vs. 59.3%). Focusing on the major hydrophilic region, the percentage of conserved residues was 81.7% in HBV + HDV HBeAg-negative infected patients and 59.2% in HBV HBeAg-negative mono-infected patients (*p* = 0.005).

Furthermore, the percentage of conserved residues showed a stable trend in patients aged <45 years, 45–55 years and >55 years in either HBV + HDV infected and HBV-mono-infected patients (Table 3), excluding the impact of patients’ age on the extent of HBsAg genetic variability.

Focusing on patients aged <45 years or >55 years, the percentage of conserved residues was higher in HBV + HDV infected than in HBV mono-infected patients (<45 years: 84.9% vs. 72.7%, *p* value = 0.006 for <45 years and 88.4% vs. 66.9%, *p* value < 0.001 for >55 years).

The HBsAg cytosolic loop II (CYL-II) encompassing the amino acid 194–201 is known to be important for the interaction of HBsAg C-terminus with the virus-signal of L-HDAg [25]. In line with this finding, the Shannon Entropy of this region was significantly lower in HBV + HDV infection than in HBV mono-infection (mean+sd: 0.04 + 0.07 in HBV + HDV infection vs. 0.20 + 0.19 in HBV mono-infection, *p* = 0.042). Furthermore, 6 out of 8 CYL-II residues were conserved (Sn = 0) in HBV + HDV infected patients while only 2 in HBV mono-infected patients (*p* = 0.046) (Figure 3 and Figure S1).

Conversely, the cytosolic loop I (CYL-I) encompassing the amino acid 56–80 is known to bind both HBV core particles and HDAg [26,27], and was conserved in both HBV + HDV infection and HBV mono-infection (21/25 conserved residues in HBV + HDV infection vs. 19/25 in HBV mono-infection) (Figure 3 and Figure S1).

### 3.3. Correlations between Specific HBsAg Mutations and the Level of Serum HDV-RNA

**Figure 4:**
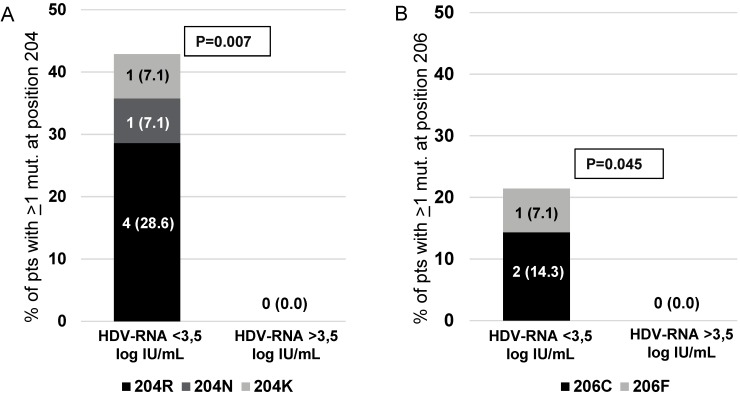
Association of mutated positions in HBsAg with levels of HDV-RNA. The histogram reports the percentage of patients with at least one mutation at HBsAg position 204 (**A**) and 206 (**B**) calculated in the group of patients with HDV-RNA <3.5 log IU/mL (*n* = 14) and with HDV-RNA >3.5 log IU/mL (*n* = 17). The prevalence of each mutation, detected at HBsAg positions 204 and 206, is reported in each bar. Statistically significant differences were assessed by Chi Squared Test based on 2 × 2 contingency table.

A further step in this study was to investigate whether the extent of genetic variability at specific HBsAg positions can affect the levels of serum HDV-RNA (reflecting HDV replicative potential and thus the burden of HDV particles produced). To achieve this goal, Shannon Entropy was calculated at each HBsAg position in the group of 14 patients with lower serum HDV-RNA (serum HDV-RNA < 3.5 log IU/mL), and in the group of 17 patients with higher serum (HDV-RNA > 3.5 log IU/mL). The HBsAg positions 204 and 206 were found conserved (Sn = 0) in the group of patients with higher serum HDV-RNA, while they were mutated only in the group of patients with lower serum HDV-RNA (% of mutated residues: 0 [0%] vs. 6 [42.9%], *p* = 0.007 for position 204; 0 [0%] vs. 3 [21.4%], *p* = 0.045 for position 206) (Figure 4). In addition, analyzing HBsAg position 207, some specific mutations were detected only in the group of patients with lower HDV-RNA and were never detected in patients with higher serum HDV-RNA (S207R/T/I: 0 vs. 6 [42.9%], *p* = 0.007).

These findings suggest that an enrichment of mutations at specific positions in HBsAg genotype D C-terminus may be detrimental for the interaction between HBsAg and the virus-assembly signal of L-HDAg, thus affecting HDV replicative potential.

### 3.4. Characterization of HDV Genotype 1 HDAg Genetic Variability

The next step of this study was to define the extent of HDAg genotype 1 genetic variability. Firstly, Shannon Entropy was calculated in the group 47 HDV genotype-1 infected patients. A lower percentage of conserved residues was observed in HDAg than HBsAg (49.5% for HDAg vs. 69.2% for HBsAg, *p* < 0.001), highlighting a lower degree of genetic conservation in HDAg than in HBsAg.

By stratifying patients HDV genotype-1 infected patients according to age (<45 years, 45–55 years and >55 years), there were no differences in terms of HDAg conserved positions (65.9%, 65.4% and 69.2%, respectively).

**Figure 5:**
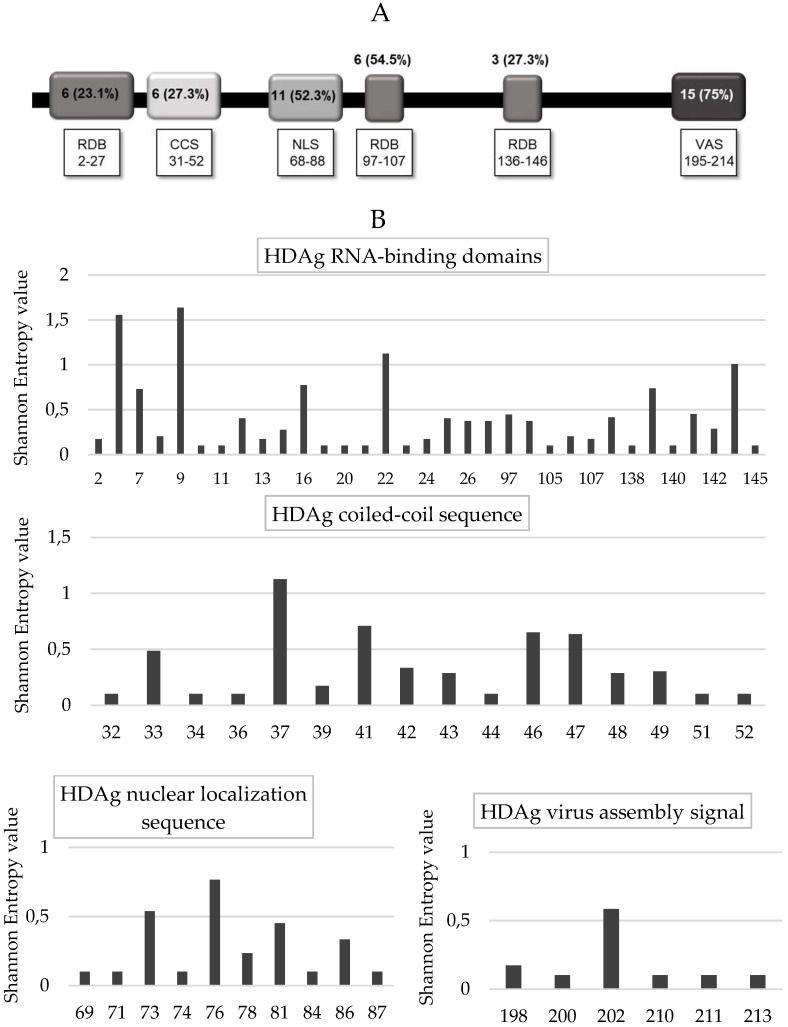
(**A**) Degree of HDAg conservation. The figure reports the number of conserved positions in each HDAg functional domains: RNA-binding domain (RBD), coiled–coil sequence (CCS), nuclear localization sequence (NLS), nuclear export signal (NES) and virus assembly signal (VAS). HDAg domains are defined according to Pascarella et al., 2010 [28]. (**B**) The histogram reports the Shannon Entropy (Sn) value at HDAg positions in 47 HDV genotype-1 infected patients. Conserved positions (Sn = 0) were not reported in the histogram. Shannon Entropy was calculated according to the following formula Sn = −Σi(pi lnpi)/lnN) where pi was the relative frequency of each distinct amino acid detected at a given position and *n* was the total number of sequences analyzed.

Furthermore, analyzing each HDAg domain, the lowest percentage of conserved residues was found in the RNA binding domain I and III, as well as in the coil–coil signal (23.1% [6/26], 27.3% [3/11], and 27.3% [6/22], respectively), followed by the RNA binding domain II (54.5%, 6/11), and the nuclear localization signal (52.3%, 11/21) (Figure 5A,B). A higher degree of conservation was found in the virus-assembly signal (aa: 195–214) than in the other HDAg domains (75% [15/20] vs. 35.2% [32/91], *p* < 0.002) (Figure 5A,B). Similarly, the mean Shannon Entropy was significantly lower in the nuclear export signal and virus-assembly signal than in the other HDV domains (mean + SD: 0.164 + 0.324 vs. 0.24 + 0.33, *p* = 0.003).

It is known that the phosphorylation of serine at HDAg positions 2 and 177 of S-HDAg increases HDV replication. In our cohort, the position 2 was conserved in 95.7% (45/47) patients, while S177 was completely conserved (Figure S2).

**Figure 6:**
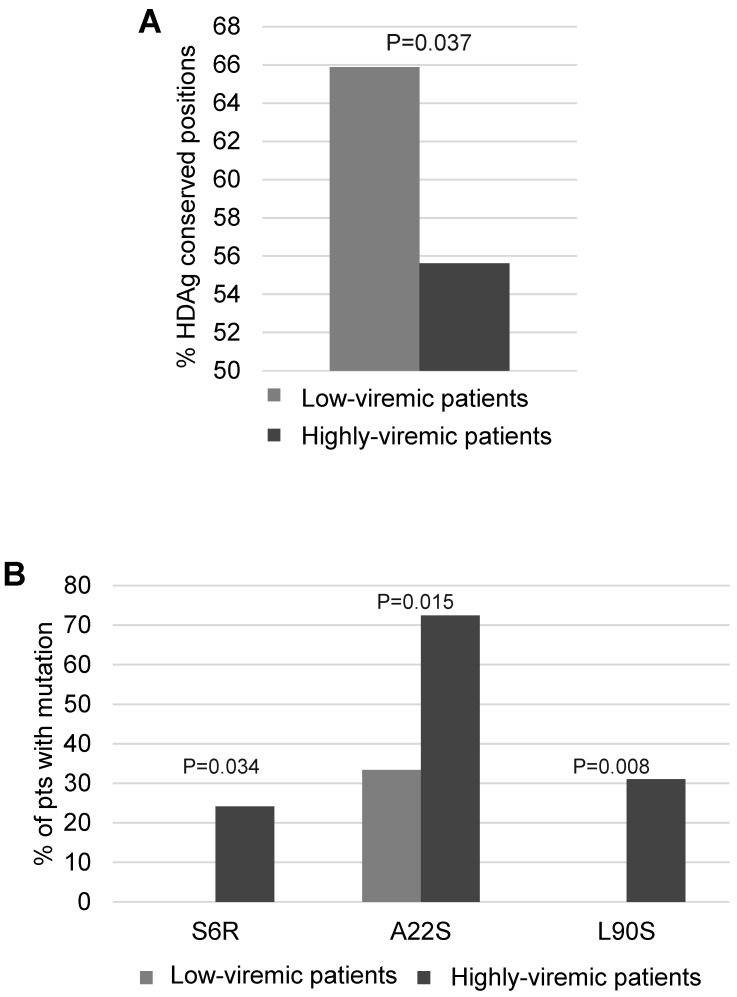
Association of mutated positions in HDAg with levels of HDV-RNA. (**A**) The histogram reports the percentage of conserved HDAg positions in patients with serum HDV-RNA <5 log IU/mL (*n* = 18) and in patients with serum HDV-RNA >5 log IU/mL (*n* = 29). (**B**) The histogram reports the percentage of patients with HDAg mutations S6R, A22S, and L90S calculated in patients with serum HDV-RNA <5 log IU/mL (*n* = 18) and in patients with serum HDV-RNA >5 log IU/mL (*n* = 29). Statistically significant difference was assessed by Chi Squared Test based on a 2 × 2 contingency table.

Then, Shannon Entropy was calculated stratifying 47 HDV genotype-1 infected patients according to serum HDV-RNA: 18 patients with serum HDV-RNA <4.5 log IU/mL, and 29 with serum HDV-RNA >4.5 log IU/mL. A lower percentage of conserved residues was observed in patients with higher serum HDV-RNA than in patients with lower serum HDV-RNA (55.6% [119/214] vs. 65.9% [141/214], *p* = 0.037) (Figure 6A). Notably, the HDAg mutations S6R, A22S and L90S occurred with significantly higher frequency in patients with higher serum HDV-RNA than in patients with lower serum HDV-RNA (S6R: 24.1% [7/29] vs. 0% [0/18], *p* = 0.034; A22S: 72.4% [21/29] vs. 33.3% [6/18], *p* = 0.015; L90S: 31% [9/29] vs. 0% [0/18], *p* = 0.008) (Figure 6B). Furthermore, the presence of at least one of these mutations was correlated with a significantly higher median (IQR) serum HDV-RNA (5.7 [5.1–6.3] log IU/mL in presence vs. 4.4 [3.8–5.6] log IU/mL in absence of S6R, A22S and L90S, *p* = 0.003). S6R and A22S are localized in RNA binding domain I, while L90S is close to nuclear localization signal (domains important for HDV life cycle). Overall findings suggest that these mutations may confer a replicative advantage to HDV genotype 1.

## 4. Discussion

This is one of the first studies showing a higher degree of genotype D HBsAg conservation in the setting of chronic HBV + HDV infection than in HBV mono-infection, highlighting that the accumulation of mutations in HBsAg in vivo can be detrimental for HDV replicative potential.

Conversely, genotype 1 HDAg was characterized by a substantially higher degree of genetic variability in most domains with the exception of the nuclear export signal and the virus-assembly domain. The identification of conserved regions in HBsAg and HDAg functional domains is crucial for the rational design of innovative anti-HDV drugs and for improving the accuracy of diagnostic assays for HDV infection.

HBsAg (particularly specific regions in HBsAg C-terminus) plays a pivotal role in HDV morphogenesis by interacting with the virus assembly signal of L-HDAg, the most conserved HDAg domain (75% of conserved residues). This supports that a higher degree of HBsAg conservation can be necessary to preserve the interaction between these two proteins, thus allowing a proper assembly and release of HDV particles. Thus, it is conceivable that HDV can exert a selective pressure on HBV favoring the production of wild-type HBsAg.

To further corroborate this concept, we found an inverse correlation between the percentage of conserved residues and levels of serum HDV-RNA.

It can be argued that the higher degree of genetic variability in HBV mono-infection may be related the collection of patients with a longer infection history. In order to address this issue, we focused the attention on HBeAg-negative patients, and we found that the percentage of conserved HBsAg residues remained higher in HBV + HDV infected patients than in HBV mono-infected patients (77.9% vs. 59.3%). Furthermore, in either HBV + HDV infected and HBV-mono-infected patients, the percentage of conserved residues showed a stable trend in patients aged <45 years, 45–55 years and >55 years. Focusing the attention on patients aged <45 years or >55 years, the percentage of conserved residues was again higher in HBV + HDV infected than in HBV mono-infected patients (<45 years: 84,9% vs. 72.7%, *p*-value 0.006 for <45 years and 88.4% vs. 66.9%, *p*-value <0.001 for >55 years).

Notably, we identified specific positions in genotype D HBsAg C-terminus (204 and 206) completely conserved in HBV + HDV infected patients with higher serum HDV-RNA (a biomarker to directly measure HDV replicative potential in vivo). Conversely, these positions were found mutated only in patients with lower serum HDV-RNA, suggesting that the presence of mutations at these positions can be detrimental for HDV replication potential. In line with this hypothesis, the positions 204 and 206 are localized in the 4th HBsAg transmembrane domain (critical to allow a proper positioning of HBsAg in the membrane of endoplasmic reticulum) [29], and in close proximity of the CYL-II (aa: 194–201) known to bind L-HDAg [25]. Previous in vitro studies have shown that mutations or deletions in HBsAg C-terminus can impair the interaction between HBsAg C-terminus and L-HDAg, thus hampering the proper envelopment of HDV ribonucleoprotein and in turn viral particles production [25]. Further studies are necessary to verify if an impairment in HBV envelopment caused by mutations in HBsAg can lead to an intracellular accumulation of HDAg thus worsening disease progression. It is also conceivable that HDV strains can further undergo a process of viral adaptation in order to preserve or increase their viral fitness. Further longitudinal studies are necessary to address HDV genetic evolution during the course of chronic infection.

Previous studies have shown that the Trp (W) at HBsAg position 196 is required for efficient assembly of stable HDV particles, for its central localization in binding interface with L-HDAg [30,31,32]. These studies showed that the mutations W196F, W196S, and W196L can abrogate HDV release [31]. Notably, due to the overlapping between the genes coding for the RT and HBsAg, W196L corresponds to the drug resistance mutation M204I. Interestingly, we detected W196L only in HBV mono-infected (8%) and never in HBV + HDV infected patients. Conversely, previous in vitro studies showed no detrimental effect on HDV release in presence of I195M (corresponding to the drug resistance mutation M204V). In line with this result, the prevalence of I195M was comparable between HBV + HDV infected and HBV mono-infected patients. These results support that HDV coinfection can constrain HBsAg evolution and modulate the emergence of drug-resistance profiles. This can have important implications for those countries in which anti-HBV drugs with lower genetic barrier are still used since M204V can also favor the emergence of cross-resistance to entecavir.

It is known that the level of serum HBV-DNA is usually lower than in HBV + HDV infected than in HBV mono-infected patients [33], and this might provide an explanation for the lower rate of HBsAg variability observed in the setting HBV + HDV infection. However, longitudinal studies have shown considerable fluctuating activity of one or both viruses with alternating predominance of HDV or HBV, highlighting a highly dynamic interplay between these two viruses [33,34].

Beyond HBV genotype D, we also detected patients infected with HBV genotype A (5, 11.9%), E (5, 11.9%), and B (1, 2.4%). Further studies are warranted in order to evaluate if the genetic background of different HBV and HDV genotypes can affect the interplay between HBV and HDV replicative potential in vivo.

In our study, we have analysed HBsAg sequences from plasma plasma. Indeed, there is a consensus on the fact that viral species circulating in patient’s plasma reflect viral species that have been originated in liver and have spread in the bloodstream. In line with this concept, we have analysed an independent set of 16 HBV chronically infected patients with concomitant plasma and liver biopsies available, and we calculated for each pair of plasma/liver sequence the genetic distance (number of different nucleotides/1000 nucleotides). We found that the genetic distance between HBsAg sequence from plasma and liver ranges from 0/1000 nucleotides to 8/1000 nucleotides (median [IQR]: 2.8 (0.3–2.8)). This supports a superimposable predominant species in plasma and liver compartments.

A different scenario was observed for HDV genotype 1 HDAg showing a significantly higher degree of variability than HBsAg.

The high degree of HDAg genotype 1 genetic variability (particularly in the small isoform) is in line with the definition of S-HDAg protein as an intrinsically disordered protein lacking of an ordered conformation. In particular, it has been proposed that the plasticity of S-HDAg may favor the interaction with a large number of cellular partners, modulating host metabolic functions, contributing to enhance viral replication and promote more acute and adverse forms of the liver disease [35]. This is in line with our results, showing the correlation between a higher degree of genetic variability and higher serum HDV-RNA.

Mechanisms underlying HDV genetic variability have not been clarified yet. HDV forces host RNA polymerase II to act as a RNA-dependent RNA polymerase to replicate viral genome. It is conceivable that the RNA polymerase II may acquire an error prone nature when RNA is used as template. As recently described, RNA polymerase II may contribute not only to the generation of point mutations but can also promote events of recombination, further enhancing HDAg genetic variability [36,37]. As postulated by recent studies, other mechanisms including ADAR1 and Apobec enzymes may represent a source of HDV genetic variability [38]. Further studies are necessary to investigate this intriguing hypothesis.

The high degree of HDAg genotype 1 genetic variability can have important clinical implications. Firstly, it can hamper HDAg recognition by the currently available serological and molecular assays for HDV diagnosis and staging [39], thus highlighting the need of a joint effort to optimize the accuracy of diagnostic assays for HDV infection. Secondly, the high degree of genetic variability might also explain the very low rate of response to interferon-based treatment. Indeed, interferon-based treatment is known to stimulate immune-response against the virus that can be hampered by the accumulation of mutations in HDAg epitopes. Longitudinal studies are necessary to investigate this point.

Among the different HDAg genotype 1 domains, the virus-assembly signal was characterized by a higher degree of genetic conservation. These regions may represent target for the design of innovative anti-HDV drugs. In line with this concept, a recent proof of concept study has shown that the prenylation inhibitor lonafarnib, acting on the virus-assembly signal can reduce serum HDV-RNA in patients with HBV + HDV infection [40,41,42].

The need of innovative anti-HDV drugs is also critical in the light of a recent study showing HDV persistence (presumably in the form of latent infection) in patients with a limited HBV replication and after liver transplantation [43]. Even more intriguingly, another study has shown that chronic HDV infection can persist in the absence of HBV replication (or when HBV replication is profoundly suppressed) if functional HBsAg is supplied from integrated HBV-DNA [44].

Furthermore, the identification of conserved HBsAg or HDAg regions can pose the basis for the design of innovative therapeutic strategies such as gene-therapy or RNA interference whose efficacy can be compromised by the accumulation of mutations in HBV or HDV genome. In particular, homing endonucleases, such as zinc-finger endonucleases (ZFNs), transcription activator-like effector nucleases (TALENs), and RNA-guided clustered regulatory interspaced short palindromic repeats associated with the Cas endonuclease family (CRISPR/Cas) have been shown to cleave selected sequences in cccDNA, thus favoring the disruption and elimination of cccDNA [45,46]. Conversely, RNA interference (by inhibiting the translation of HBV proteins) can remarkably decrease the burden of viral antigens (including HBsAg) circulating in patients’ sera (Flisiak et al., Expert Opin Biol Ther. 2018) [47]. These approaches have been so far investigating in HBV, further studies are necessary to investigate their role in counteracting HDV replication.

## 5. Conclusions

In conclusion, genotype D HBsAg genetic conservation was significantly higher in the setting of chronic HBV + HDV infection than in HBV mono-infection, suggesting HDV role in constraining HBV genetic evolution in order to preserve HDV fitness. Conversely, genotype 1 HDAg is characterized by a higher degree of genetic variability with the exception of the nuclear export signal and the virus-assembly signal. The identification of conserved regions in HBsAg and HDAg is crucial for the rational design of innovative anti-HDV drugs and for improving the accuracy of diagnostic assays of HDV infection.

## Figures and Tables

**Table 1:** Characteristics of 78 patients with HBV + HDV infection.

Characteristics	
Male, *n* (%)	51 (65.4%)
Italian, *n* (%)	42 (53.8%)
Median age (IQR), year	51 (41–59)
Median AST, IU/L (IQR)	66 (34–101)
Median ALT, IU/L (IQR)	68 (43–127)
Median serum HBV-DNA, log IU/mL (IQR)	2 (1.3–3.1)
Median serum HDV-RNA, log IU/mL (IQR)	4.7 (2.5–6.1)
**Current Treatment**	
NUCs, *n*( %) ^a^	33 (50%)
**Liver disease**	
Cirrhosis, *n* (%)	20 (28.2%)
Hepatocellular carcinoma, *n* (%)	7 (9.3%)

^a^ Therapy status (drug-naïve/drug-treated) was available for 66 patients; Abbreviations: NUC, nucleos(t)ide reverse transcriptase inhibitors; ALT, alanine-aminotransferase; AST aspartate aminotransferase.

**Table 2:** Characteristics of 31 HBV + HDV infected and 62 HBV mono-infected patients.

Characteristics	HBV + HDV Infection	HBV Mono-Infection	*p*-Value
Male, *n* (%)	19 (61.3)	41 (66.1)	0.653
Italian, *n* (%)	18 (58.1)	41 (66.1)	0.498
Median age (IQR), year	51 (41–59)	49 (38–59)	0.554
Median HBV-DNA (IQR), log IU/mL	3.0 (1.8–3.4)	3.1 (2.5–3.9)	0.147
Median ALT (IQR), IU/L ^a^	55 (28–94)	35 (25–52)	0.088
Median AST (IQR), IU/L ^a^	46 (26–61)	26 (17–39)	0.004
Drug-Naïve, *n* (%)	17 (54.8)	37 (59.7)	0.626

^a^ Median ALT and AST (IQR) was calculated on 47/62 HBV mono-infected patients and 29/31 HBV + HDV coinfected patients with the datum available.

**Table 3:** Percentage (*n*) of HBsAg conserved residues by stratifying patients according to age ^a^.

	HBV + HDV Infected Patients ^c^				HBV Mono-Infected Patients ^d^			
Amino Acids Positions	<45 Years	45–55 Years	>55 Years	*p* Value ^b^	<45 Years	45–55 years	>55 Years	*p* Value ^b^
55–98 aa	97.7 (43)	97.7 (43)	93.2 (41)	0.221	88.6 (39)	86.4 (38)	84.1 (37)	0.117
99–169 aa	85.9 (61)	83.1 (59)	88.7 (63)	0.602	78.9 (56)	83.1 (59)	70.4 (50)	0.602
170–226 aa	73.7 (42)	70.2 (40)	84.2 (48)	0.602	52.6 (30)	70.2 (40)	49.1 (28)	0.602
total 55–226 aa	84.9 (146)	82.6 (142)	88.4 (152)	0.602	72.7 (125)	79.7 (137)	66.9 (115)	0.602

^a^ The % was calculated on the total number of residues within each HBsAg domain; ^b^ *p* values were calculated by Chi Squared test for trend; ^c^ HBV + HDV coinfected patients were stratified according to age: <45 years (*n* = 10), 45–55 years (*n* = 13), and > 55 years (*n* = 8); ^d^ HBV mono-infected patients were stratified according to age: <45 years (*n* = 26), 45–55 years (*n* = 16), and > 55 years (*n* = 20).

## Supplementary Materials

The following are available online at http://www.mdpi.com/1999-4915/10/7/363/s1. Figure S1: Frequency of amino acid substitutions detected at each HBsAg genotype-D position. Figure S2: Frequency of amino acid substitutions detected at each HDAg genotype-1 position.
