# Supplementary material for: HDV Can Constrain HBV Genetic Evolution in HBsAg: Implications for the Identification of Innovative Pharmacological Targets
Source: Viruses. 2018 Jul 9;10(7):363. doi: 10.3390/v10070363 (PMC6071122; doi:10.3390/v10070363)
Supplement: Supplementary file 1 [file viruses-10-00363-s001.pdf]

A. HBV+HDV Infection

N-terminus

S55	P56	T57I$_1$	S58	N59	H60	S61	P62	T63	S64	C65	P66	P67	T68	C69
P70	G71	Y72	R73H$_1$	W74	M75	C76Y$_1$	L77	R78	R79H$_1$	F80	I81	I82	F83	L84
F85	I86	L87	L88	L89	C90	L91	I92	F93	L94S$_1$	L95	V96	L97	L98	

Major hydrophilic region

D99	Y100F$_1$	Q101H$_2$	G102	M103	L104	P105	V106	C107	P108	L109	I110L$_1$	P111	G112R$_1$S$_1$	S113
S114	T115	T116N$_1$	S117	T118A$_1$	G119	P120T$_1$	C121	R122	T123A$_2$	C124Y$_1$	T125M$_5$	T126S$_1$	P127T$_6$	A128
Q129	G130	T131N$_1$P$_1$	S132	M133I$_1$	Y134F$_1$N$_1$S$_1$	P135	S136Y$_1$	C137	C138	C139	T140I$_1$	K141	P142	S143
D144	G145	N146	C147	T148	C149	I150	P151	I152	P153	S154	S155	W156	A157	F158
G159A$_2$R$_2$I$_1$V$_1$	K160	F161	L162	W163	E164D$_2$A$_1$G$_1$	W165	A166	S167	A168	R169P$_1$				

C-terminus

F170	S171	W172*$_1$L$_1$	L173P$_1$	S174N$_1$	L175S$_2$	L176	V177	P178	F179	V180	Q181	W182	F183	V184A$_1$
G185E$_1$	L186R$_2$	S187	P188	T189I$_2$	V190	W191*$_1$	L192	S193L$_4$	V194A$_1$	I195M$_1$	W196	M197	M198	W199
Y200	W201	G202	P203R$_2$	S204R$_4$N$_1$K$_1$	L205	Y206C$_2$F$_1$	S207R$_3$N$_2$T$_2$I$_1$	I208T$_3$	L209S$_1$	S210	P211R$_2$	F212L$_1$	L213I$_4$	P214L$_2$
L215	L216*$_3$	P217L$_4$	I218	F219	F220L$_1$	C221Y$_1$	L222R$_1$	W223	V224I$_1$A$_1$	Y225	I226			

B. HBV Mono-Infection

N-terminus

S55C$_5$	P56	T57	S58	N59	H60	S61	P62	T63	S64Y$_1$	C65	P66	P67	T68I$_1$	C69*$_3$
P70	G71	Y72	R73	W74	M75	C76W$_2$Y$_2$	L77V$_1$	R78	R79	F80S$_2$	I81	I82	F83S$_1$	L84F$_1$
F85Y$_1$	I86	L87	L88	L89	C90	L91	I92T$_3$	F93	L94	L95	V96A$_1$	L97R$_1$	L98	

Major hydrophilic region

D99	Y100C$_1$	Q101	G102	M103T$_1$	L104	P105A$_1$	V106A$_1$	C107C*$_1$	P108	L109	I110L$_1$M$_1$	P111	G112R$_2$E$_2$N$_1$S$_1$D$_1$K$_1$	S113
S114T$_1$	T115A$_1$	T116	S117W$_1$G$_1$	T118K$_2$V$_2$A$_1$	G119	P120S$_2$A$_1$T$_1$	C121	R122K$_1$	T123	C124	T125M$_7$	T126S$_1$I$_1$	P127T$_{12}$S$_1$A$_1$	A128V$_2$
Q129P$_1$	G130	T131N$_2$	S132	M133L$_1$T$_1$	Y134N$_2$F$_2$S$_2$	P135	S136	C137	C138	C139	T140	K141	P142L$_1$	S143T$_1$
D144	G145R$_2$	N146	C147Y$_1$	T148	C149	I150T$_1$	P151	I152	P153	S154	S155	W156	A157	F158L$_1$
G159A$_4$V$_1$	K160S$_1$N$_1$	F161Y$_2$	L162	W163	E164G$_4$D$_1$V$_1$	W165	A166	S167	A168T$_1$	R169H$_1$				

C-terminus

F170	S171C$_1$	W172C$_2$S$_1$*$_1$	L173	S174N$_1$	L175S$_2$	L176	V177A$_4$L$_1$	P178	F179	V180	Q181	W182	F183C$_2$	V184A$_2$
G185E$_1$	L186P$_2$C$_1$A$_1$V$_1$	S187	P188	T189I$_6$	V190	W191	L192P$_1$	S193L$_3$	V194A$_3$I$_1$	I195M$_5$T$_1$	W196L$_2$	M197T$_2$	M198I$_1$	W199
Y200F$_6$C$_6$	W201	G202	P203Q$_1$R$_1$L$_1$	S204N$_7$T$_2$R$_1$I$_1$	L205P$_1$	Y206C$_8$H$_4$F$_2$L$_1$	S207N$_{14}$R$_{11}$T$_6$H$_1$I$_1$	I208T$_6$	L209W$_1$V$_1$	S210R$_9$K$_2$N$_1$	P211H$_2$	F212L$_1$S$_1$	L213I$_9$	P214Q$_1$L$_1$
L215	L216*$_3$F$_1$S$_1$	P217L$_6$A$_1$S$_1$	I218	F219S$_1$	F220L$_6$C$_1$Y$_1$	C221F$_1$Y$_1$	L222	W223	V224A$_1$	Y225F$_2$	I226			

No mutation	0.1-5%	6-20%	>20%

Figure S1. Frequency of amino acid substitutions detected at each HBsAg genotype-D position. HBsAg genotype-D protein is colored according to the frequency of mutations observed at each HBsAg position in HBV+HDV infected patients (A) and HBV mono-infected patients (B). Wild-type amino acid is reported on the left while mutated amino acid on the right of each position. The absolute frequency of each mutation is reported in subscript.

RBD start

M1	$S2G_2$	R3	S4	E5	$S6E_7R_7K_6G_6A_2{*}_2L_1$	$R7K_{31}G_1$	$K8R_1E_1$	$N9D_9K_6I_3S_3Y_1F_1G_1H_1T_1$	$R10S_1$	$G11R_1$
$G12R_1N_1D_1A_1V_1$	$R13K_2$	E14	$E15D_2K_1$	$I16V_{26}L_1$	L17	E18	$Q19E_1$	$W20R_1G_1$	$V21I_1$	$A22S_{27}N_{16}T_3I_2G_1V_1$
$G23A_1$	$R24KKL_{del}$	$K25R_3$	$K26R_1Q_1$	$L27Q_1S_1$	E28	$E29D_1$	L30			

CCS start

E31	$R32K_1$	$D33E_9$	$L34V_1$	R35	$K36R_1$	$T37V_{28}A_6I_2M_1$	K38	$K39R_2$	K40	$L41I_{38}V_4M_1$
$K42R_3G_1$	$K43R_4$	$L44F_1$	E45	$D46E1_7$	$E47D_{36}G_1$	$N48H_4$	$P49L_1A_1S_1$	W50	$L51P_1$	$G52E_1$
$N53D_1$	$I54V_9$	K55	G56	I57	$L58I_4$	G59	K60	$K61R_1$	$D62N_2E_1$	$K63R_9$
$D64G_3$	G65	$E66G_4$	G67							

NLS start

A68	$P69L_1$	P70	$A71S_1$	K72	$R73K_{11}$	$A74P_1$	R75	$T76V_5M_2A_2S_1$	D77	$Q78R_3$
M79	E80	$V81I_8$	D82	S83	$G84S_1$	P85	$R86G_5$	$K87R_1$	R88	$P89R_1$
$L90S9A_2F_1T_1$	$R91G_1$	G92	G93	F94	T95	$D96N_1$				

RBD start

$K97Q_3R_2E_1$	E98	R99	$Q100E_2R_1K_1$	D101	H102	R103	R104	$R105K_1$	$K106R_1M_1$	$A107N_1T_1D_1$

RBD start

$E136D_7$	D137	$E138K_1$	$R139K_6E_6$	$R140G_1$	$E141K_8$	$R142G_4$	R143	$V144I_{11}T_3A_2L_1$	$A145T_1$	G146

VAS start

P195	W/*196	D197	I198	L199	$F200L_1F_1$	P201	$A202S_{13}$	D203	P204	P205
F206	S207	P208	Q209	$S210G_1$	$C211S_1$	R212	$P213T_1$	Q214		

No mutation	0.1-5%	6-20%	20-50%	>50%

Figure S2. Frequency of amino acid substitutions detected at each HDAg genotype-1 position. The sequence of HDAg (1-214 aa) is colored according to the frequency of mutations observed at each position of HDAg functional domains in 47 plasma samples from HBV+HDV infected patients (all infected with HDV genotype 1). Wild-type amino acid is reported on the left while mutated amino acid on the right of each position. The absolute frequency of each mutation is reported in substrict. * indicates the presence of stop codon at a given position.
